# RNA Therapeutics - Research and Clinical Advancements

**DOI:** 10.3389/fmolb.2021.710738

**Published:** 2021-09-22

**Authors:** Rundong Feng, Suryaji Patil, Xin Zhao, Zhiping Miao, Airong Qian

**Affiliations:** ^1^ Shaanxi Institute for Food and Drug Control, Xi’an, China; ^2^ Lab for Bone Metabolism, Xi’an Key Laboratory of Special Medicine and Health Engineering, Key Lab for Space Biosciences and Biotechnology, Research Center for Special Medicine and Health Systems Engineering, NPU-UAB Joint Laboratory for Bone Metabolism, School of Life Sciences, Northwestern Polytechnical University, Xi’an, China; ^3^ School of Pharmacy, Shaanxi Institute of International Trade & Commerce, Xi’an, China

**Keywords:** RNA-based therapy, siRNA, antisense oligonucleotides, aptamers, CRISPR-Cas, clinical trial, FDA approved

## Abstract

RNA therapeutics involve the use of coding RNA such as mRNA as well as non-coding RNAs such as small interfering RNAs (siRNA), antisense oligonucleotides (ASO) to target mRNA, aptamers, ribozymes, and clustered regularly interspaced short palindromic repeats-CRISPR-associated (CRISPR/Cas) endonuclease to target proteins and DNA. Due to their diverse targeting ability and research in RNA modification and delivery systems, RNA-based formulations have emerged as suitable treatment options for many diseases. Therefore, in this article, we have summarized different RNA therapeutics, their targeting strategies, and clinical progress for various diseases as well as limitations; so that it might help researchers formulate new and advanced RNA therapeutics for various diseases. Additionally, U.S. Food and Drug Administration (USFDA)-approved RNA-based therapeutics have also been discussed.

## Introduction

The Human Genome Project (HGP) has revealed vast information about the human genome and has greatly enhanced its role in the development of biomedical research. (Lander et al., 2001; Wan et al., 2014; Lekka and Hall, 2018) As a result of advancements in next-generation sequencing technology, researchers have been able to reveal the role of some genetic factors in many diseases such as cancer, rheumatoid arthritis, Parkinson’s, and Alzheimer’s disease. (Kaczmarek et al., 2017) Moreover, the results of many studies have revealed the important role of coding as well as non-coding RNAs (ncRNAs), such as microRNAs (miRNA), long ncRNA (lncRNA), circular RNA (circRNA), and small interfering RNAs (siRNAs) in various diseases. (Gao et al., 2020) This has provided potential insight into developing possible treatments of various diseases by introducing nucleic acids into the cell to control the expression of altered genes permanently or transiently. (Smith and Blomberg, 2017) However, due to the inherent instability of RNA, it is often required to be delivered to the target site, which can be addressed by improving delivery systems through various modifications. (Patil et al., 2019) As a result, many RNA-based therapeutics involving siRNAs, ASO, ribozymes, mRNA, aptamers, and CRISPR/Cas have been developed and are being tested for their potential as a possible intervention strategy in various diseases (Moss et al., 2019) such as heart diseases, neurological diseases (amyotrophic lateral sclerosis (ALS), Alzheimer’s), and cancers. (Bekris and Leverenz, 2015; Reddy and Miller, 2015; De Majo and De Windt, 2018; Lei et al., 2019) In this review, we have summarized the clinical progress of siRNA, RNA ASO, RNA aptamers, ribozyme, mRNAs, and CRISPR guide RNAs (gRNAs) as RNA therapeutics to control gene expression through RNA, DNA, and protein as well as their limitation. In addition, the currently USFDA-approved RNA-based drugs and their future potential are also discussed.

## RNA Therapeutics

The strategies for RNA therapeutics involve the use of both, coding as well as non-coding RNAs. There are five types of therapeutic RNAs, *1*) RNAs that inhibit RNA activity. This includes the use of siRNAs, antisense RNAs, *2*) RNAs that target proteins such as RNA aptamers, *3*) RNAs that reprogram genetic information including trans-splicing ribozyme, *4*) RNAs that encode therapeutic proteins (mRNAs), and *5*) DNA modifying CRISPR guide RNAs (gRNAs) (Sullenger and Nair, 2016).

### SiRNA

RNA interference (RNAi) involves the use of siRNAs to control gene function. These are long double-stranded RNA (dsRNA) molecules with a characteristic 3′ overhang. (Aagaard and Rossi, 2007) SiRNAs are generated by the ribonuclease Dicer through endonucleolytic processing, which is an endonuclease belonging to the RNase III family to produce ∼21–25 nucleotide dsRNA. (Gavrilov and Saltzman, 2012) Once produced, Dicer transfers siRNAs to the RNA-induced silencing complex (RISC), which houses the Argonaute 2 that degrades target mRNA molecules. (Aagaard and Rossi, 2007) Because of this ability of siRNA, they have been investigated as a possible therapeutic. But given their single-stranded nature, their efficacy is limited. However, the modifications to siRNA have assisted in improving the safety, stability, efficiency, and specificity of siRNA. (Selvam et al., 2017; Saw and Song, 2020) Moreover, sRNAs synthesized by biological methods can provide stability. The biological method involves the use of T7 phage RNA polymerase to transcribe a siRNA from short double-stranded oligo cassettes enclosing the promoter sequence. The strands are produced in a separate reaction and hybridized before they can be purified. However, this method often produces siRNAs containing a promoter-derived GGG sequence and a 5′ triphosphate group, which, if left as is, activates non-specific gene expression inhibition *via* the interferon pathway. Thus, the hybridized siRNA is processed by T1 ribonuclease to remove the single-stranded 5′ GGG overhang. (Amarzguioui et al., 2005) The chemical methods, such as the introduction of 2′-deoxy-2′-fluorouridine or locked nucleic acid (LNA) nucleotides, 2′-O-allylation 2′-O-methylation, or phosphorothioates, have advantages in terms of uniform composition, higher rate of production, and enhanced thermal stability without affecting RNAi efficiency. (Amarzguioui et al., 2003; Braasch et al., 2003) To achieve long-term gene silencing, expression cassettes coding engineered siRNA can be efficiently used. Expression cassettes are made up of a promoter, the gene of interest, and a terminator and are introduced into the cell using delivery systems. Type 3 RNA polymerase III (Pol III) promoter is most common choice of promoter and structural or catalytic RNAs are encoded by Pol III genes. RNA molecules expressed from U6 or H1 Type 3 Pol III promoters then mediate sequence-specific RNAi-based gene inhibition. (Ill and Chiou, 2005; Marín-garcía and MarÍN-GarcÍA, 2007; Do et al., 2019) A list of siRNA therapeutics can be seen at Table 1.

**TABLE 1:** siRNA therapeutic applications in clinical trials.

Name	Disease	Target	ClinicalTrials.gov Identifier	Recruitment Status	Phase	Reference
Inclisiran	Homozygous Familial Hypercholesterolemia	PCSK9	NCT02597127	Completed	II	Ray et al. (2017)
			NCT03397121	Completed	III	Raal et al. (2020)
AGN211745	CNV-AMD	VEGFR1	NCT00363714	Completed	I & II	Kaiser et al. (2010)
PF-04523655	CNV-AMD	VEGFR1	NCT00713518	Completed	II	Nguyen et al. (2012a)
PF-04523655	Diabetic Macular Edema	RTP801	NCT00701181	Terminated	II	Nguyen et al. (2012b)
QPI-1007	Non-Arteritic Anterior Ischemic Optic Neuropathy	Caspase 2	NCT01064505	Completed	I	Antoszyk et al. (2013)
siG12D LODER	Pancreatic Cancer	KRASG12D	NCT01188785	Completed	I	Golan et al. (2015)
			NCT01676259	Unknown	II	Varghese et al. (2020)
TKM-080301	Hepatocellular Carcinoma	PLK1	NCT02191878	Completed	I/II	El Dika et al. (2019)
	Solid Cancer		NCT01262235	Completed	I/II	Demeure et al. (2016)
Atu027	Advanced Solid Tumors	Protein kinase N3	NCT00938574	Completed	I	Schultheis et al. (2014)
	Pancreatic Cancer		NCT01808638	Completed	I/II	Schultheis et al. (2016)
DCR-MYC	Solid Tumors, Multiple Myeloma, or Lymphoma	MYC	NCT02110563	Terminated	I	Tolcher et al. (2015)
CALAA-01	Solid Tumor	RRM2	NCT00689065	Terminated	I	Davis et al. (2010)
TD101	Pachyonychia congenita	KRT6A	NCT00716014	Completed	I	Leachman et al. (2010)
ARC-520	Healthy	cccDNA-derived viral mRNA	NCT01872065	Completed	I	Schluep et al. (2017)
	Chronic Hepatitis B		NCT02452528	Terminated	II	Yuen et al. (2020)
QPI-1002	Acute Renal Failure	p53	NCT00554359	Completed	I	Demirjian et al. (2017)
	Acute kidney injury		NCT00802347	Completed	I/II	Peddi et al. (2014)

Familial hypercholesterolemia, which is characterized by a higher level of low-density lipoprotein-cholesterol (LDL-C), increases the risk of atherosclerotic cardiovascular disease (CD). (Raal et al., 2020) The LDL receptor (LDLR) and bound LDL are transported to the endosomes, where LDL is degraded to amino acids and cholesterol, while LDL-R is recycled. The proprotein convertase subtilisin/Kexin type 9 (PCSK9) binds to the LDL receptors (LDL-R) on hepatocytes and promotes its degradation. Therefore, it is essential to block PCSK9 to stimulate LDL-R recycling to lower blood LDL concentrations. (Ciccarelli et al., 2018) A subcutaneous injection of inclisiran, a formulation of phosphorothioate, 2′-O-methyl nucleotide, and 2′-fluoro nucleotide modified-siRNA directed against PCSK9 conjugated to triantennary N-acetylgalactosamine carbohydrates, has demonstrated to target and lower PCSK9 levels and LDL cholesterol levels in patients with high cardiovascular risk. (Ray et al., 2017; Raal et al., 2020) Many studies conducted using siRNA for choroidal neovascularization caused due to age-related macular degeneration (CNV-AMD) to target vascular endothelial growth factor receptor-1 (VEGFR1) or RTP801, have shown promising results in various phases of clinical trials. (Nguyen et al., 2012a; Nguyen et al., 2012b) In optic nerve atrophy studies focusing on QPI-1007 (a chemically altered siRNA to inhibit caspase 2 expressions) showed good tolerability and improved visual acuity (VA) in patients. (Antoszyk et al., 2013; Solano et al., 2014)

#### SiRNA Therapeutics in Cancer

The metastasis and invasion are major processes that lead to the proliferation of cancers. Many cancer drugs require their long-term use to be effective and often produce unwanted side effects. SiRNA therapeutics are being tested for the prevention or treatment of various cancers. KRAS mutation is the most well-known alteration in various cancers. The study (NCT01188785) of siG12D-LODER, a biodegradable polymeric matrix enclosing a siRNA against KRASG12D, has demonstrated that the polymer was able to target the tumor and reduce its progression. (Golan et al., 2015) Further investigation (NCT01676259) is underway to test the efficacy of siG12D-LODER in combination with chemotherapeutic agents such as gemcitabine and nab-paclitaxel in locally advanced pancreatic cancer patients. (Varghese et al., 2020) Polo-like kinases (Plks) are involved in cell cycle regulation and cell proliferation. Plk is a group of five serine/threonine kinases and is often overexpressed in cancer cells. (Goroshchuk et al., 2019) Inhibition of Plk is known to reduce cancer cell proliferation. Therefore, TKM-080301, a lipid nanoparticle (LNP) formulation made up of four lipids and a synthetic, double‐stranded siRNA targeting human PLK1 mRNA was prepared and tested. The intravenous infusion of TKM-080301 showed good tolerability but demonstrated a limited antitumor effect and no overall survival effect in patients with advanced hepatocellular carcinoma. (El Dika et al., 2019) However, TKM-080301 showed a 13% reduction in tumor diameter in adrenocortical cancer (ACC). (Demeure et al., 2016)

Protein kinase N3 (PKN3) is a downstream effector of the phosphoinositide-3-kinase (PI3K) pathway and its inhibition in vascular and lymphatic endothelial cells suppresses tumor progression and lymph node metastasis. (Hattori et al., 2017) Atu027, a liposomal PKN3 siRNA formulation, showed no safety issues in patients with advanced solid tumors and at the end of treatment stabilized disease condition in 41% of the participant. (Schultheis et al., 2014) In the subsequent study, a combination of Atu027 and gemcitabine in locally advanced or metastatic pancreatic adenocarcinoma displayed safety and good acceptability. (Schultheis et al., 2016)

In several human cancer tumorigeneses, Myc oncoprotein is known to play a critical role and inhibition of Myc significantly reduce tumor cell growth as well as proliferation. (Ponzielli et al., 2005) Accordingly, a therapeutic, DCR-MYC, a synthetic double-stranded RNA in a stable lipid particle suspension directed against MYC, has been developed and its intravenous infusion in solid and hepatocellular carcinoma patients showed good tolerability and encouraging early clinical and metabolic responses. (Tolcher et al., 2015) Furthermore, during cancer, phenotypes related to malignancy are often associated with increased levels of phosphorylation of tyrosine protein. Ephrin type-A receptor 2 (EPHA2), a member of receptor tyrosine kinases is predominantly expressed in epithelial cells and elevated levels drive the malignant transformation and tumorigenic ability in mammary epithelial cells. (Zelinski et al., 2001) In preclinical mammalian studies, multiple doses of EPHARNA, an EphA2 siRNA capsulated in dioleoylphosphatidylcholine (DOPC) nanoliposome were tested in mice and *Rhesus* macaques. It demonstrated no pathological or dose-related microscopic findings however, stimulated a mild to moderate inflammatory response and mild hemolytic effect in the female mice while minimal to moderate infiltration of mononuclear cells in *Rhesus* macaques. (Wagner et al., 2017) The level of Ribonucleoside-diphosphate reductase subunit M2 (RRM2) is increased in cancer to ensure a continuous supply of 2′-deoxyribonucleoside 5′-triphosphates (dNTPs) during DNA replication. (Morikawa et al., 2010) Therefore, targeting RRM2 with siRNA could have therapeutic potential in melanoma patients. A therapeutic formulation, CALAA-01, was synthesized by encapsulating RRM2 siRNA in cyclodextrin-adamantane polyethylene glycol (AD-PEG)- human transferrin protein (hTf). The systemic administration of CALAA-01 not only reduced levels of RRM2 mRNA and the protein but was also efficient localized inside the tumor (Davis et al., 2010).

#### SiRNA Therapeutics in Other Diseases

The autosomal recessive primary hyperoxalurias (PHs) are characterized by increased overproduction of oxalate in the liver due to the lack of metabolic enzymes. (Milliner, 2016) Overproduction of oxalate leads to the production of insoluble calcium oxalate (CaOx) crystals in the kidney, causing renal failure. In primary hyperoxaluria type 1 (PH1), which is caused by mutations in alanine-glyoxylate aminotransferase (AGT), siRNA therapy in the mice and non-human primates has shown potential in reducing the expression of glycolate oxidase (GO) by targeting GO mRNA. The reduced expression of GO led to stabilized urine oxalate levels and reduced CaOx deposition in a preclinical PH1 mouse model (Dutta et al., 2016).

The chronic viral infection of hepatitis B virus (HBV) can develop into chronic hepatitis, cirrhosis, and hepatocellular carcinoma (HCC) and covalently closed circular DNA (cccDNA) integrated into the nucleus of hepatocytes persistently produces transcripts that serve as templates for the translation of HBV early antigen (HBeAg) and surface antigens (HBsAg) proteins, which are important in the production of new viral particles. Therefore, HBeAg and HBsAg are targeted for new therapies. (Yuen et al., 2020) ARC-520 was developed by conjugating two synthetic siRNAs against (cccDNA)-derived viral mRNA of hepatitis B to cholesterol as a siRNA therapeutic for chronic hepatitis B virus infection. In healthy volunteers, it showed good tolerability but stimulated mast cell degranulation-mediated histamine release. (Schluep et al., 2017) In another study, the effect of ARC-520 on a reduction in hepatitis B surface antigen (HBsAg) was evaluated with good tolerability. (Yuen et al., 2020) In liver and idiopathic pulmonary fibrosis, lipid nanoparticle encapsulated heat shock protein 47 (HSP47) siRNA (ND-L02-s0201/BMS-986263) showed a significant reduction in lung weight as well as fibrosis scores and considerable enhancement in lung function (Zabludoff et al., 2017).

Acute kidney injury (AKI) is generally treated by supportive treatments due to the absence of approved therapeutic agents. P53 is known to play an important role in ischemia reperfusion-induced AKI and has emerged as the pivotal regulator of apoptosis and p53 inhibition in several ischemia-reperfusion injury models has demonstrated to enhance renal function post-injury and improve histology. A clinical study evaluating QPI-1002 (containing I5NP, a siRNA against p53) has shown that intravenous injection was safe and well-tolerated by AKI patients. (Demirjian et al., 2017) Furthermore, QPI-1002 (NCT00802347) also reduced delayed graft function (DGF) during kidney transplants and considerably enhanced the time to the first dialysis (Peddi et al., 2014).

The beta-thalassemia or hereditary hemochromatosis are described by iron accumulation due to dysregulation in the hepcidin-ferroportin axis, regulated by transmembrane protease matriptase-2 that is encoded by the TMPRSS6 gene, causes organ damage and severe clinical complications. (Altamura et al., 2018) In the preclinical study, GalNAc-TMPRSS6 siRNA conjugate, SLN124, was able to decrease systemic and tissue iron levels in a hereditary hemochromatosis type 1 mice model. The beta-thalassemia intermedia model showed long-term effects on target gene expression and the iron store modulation and stabilized erythropoiesis and anemia (Altamura et al., 2018; Altamura et al., 2019).

### ASO

ASOs are 18–30 base pairs, single-stranded RNA/DNA molecules designed to specifically inhibit mRNA function. They bind to a specific mRNA with Watson-Crick base-pairing, impairing mRNA translation or degrading mRNA through RNase H. (Atri et al., 2019; Scoles et al., 2019) Apart from RNase H and altering splicing, other mechanisms of action of ASO include steric hindrance, destabilization of pre-mRNA, and miRNAs cleavage to control gene expression (Chi et al., 2017).

Here, we have focused on RNA ASOs designed for various diseases. For the treatment of eye diseases many pre-clinical and clinical trials have shown promising results. Leber congenital amaurosis (LCA) retinal dystrophy is linked with juvenile blindness or severe visual impairment due to intronic mutation in various genes including centrosomal protein 290 (CEP290). (den Hollander et al., 2008) QR-110, a single-stranded, phosphorothioated, 2′O-methyl-modified splice-modulating RNA oligonucleotide was developed to target CEP290. The intravitreal injection of QR-110 (NCT03140969 and NCT03913143) showed that modified ASO was able to re-establish the levels of CEP290 in LCA10 primary fibroblasts *in vitro* and localize in the retinal layers in mice and rabbits. In monkeys, the administration was favorably tolerated, correcting the splicing defect, demonstrating efficacy and safety (Dulla et al., 2018).

### Aptamers

Aptamers are single-stranded nucleic acid (DNA or RNA) molecules that bind and inhibit proteins. (Nimjee et al., 2017) The ability of aptamers to form shapes offers high affinity as well as excellent specificity toward targets. (Adachi and Nakamura, 2019) Because of their similar modes of action to antibodies and synthetic origin, aptamers are also called chemical antibodies. The method known as systemic evolution of ligands by exponential enrichment (SELEX) is used to produce aptamers *in vitro* and used not only for detection, inhibition but also for the characterization of specific targets. (Banerjee and Nilsen-Hamilton, 2013) Various aptamers (Table 2) have entered the clinical trials for various diseases such as macular degeneration, diabetic macular edema, and chronic inflammatory diseases.

**TABLE 2:** Aptamer in clinical trials.

Name	Disease	Target	ClinicalTrials.gov Identifier	Recruitment Status	Phase	Reference
Zimura	Macular Degeneration	C5	NCT02686658	Completed	II/III	Jaffe et al. (2020)
Pegcetacoplan	Geographic atrophy	C3	NCT02503332	Completed	II	Liao et al. (2020)
E10030	Macular Degeneration	PDGF	NCT00569140	Completed	I	Jaffe et al. (2016)
E10030	Macular Degeneration	PDGF	NCT01089517	Completed	II	Jaffe et al. (2017)
ARC1779	von Willebrand Disease	von Willebrand Factor (vWF) A1	NCT00432770	Completed	I	Gilbert et al. (2007)
NOX-H94	Anemia of Chronic Disease	Hepcidin	NCT01691040	Completed	II	Georgiev et al. (2014)
NOX-E36	Type 2 Diabetes Mellitus, Albuminuria	C-C motif-ligand 2	NCT01547897	Completed	II	Menne et al. (2017)

AMD, a common macular disease is a multifactorial disorder and a prominent cause of visual impairment and vision loss. It is characterized by the presence of drusen in the early stage of neovascularization and atrophy in the late stage. (Mitchell et al., 2018) A study has shown that vascular endothelial growth factor (VEGF) contribute significantly in the progression of several ocular pathologies involving neovascularization. (Andreoli and Miller, 2007) Thus, anti-VEGF therapy has been developed for macular degeneration and diabetic macular edema. EYE001, a pegylated aptamer to VEGF, has shown the ability to completely reduce the vascular leakage mediated by VEGF, neovascularization in corneal angiogenesis model, as well as 80% inhibition in retinal neovascularization, resulting in improved vision. (Martinet al., 2002) The studies on multiple-dose safety of EYE001 alone or in conjunction with photodynamic therapy in subfoveal CNV secondary to AMD patients showed stabilized or improved vision. (The Eyetech Study Group, 2003) Apart from VEGF, the complement pathway also plays an important role in AMD. (Sivaprasad and Chong, 2006) The complement system targeting inhibitors, such as Zimura, a polyethylene glycol (PEG), chemically synthesized single-stranded nucleic acid aptamer targeting complement factor C5 and pegcetacoplan, a synthetic cyclic peptide conjugated to a polyethylene glycol (PEG) that specifically target C3 and C3b have shown capacity to decrease geographic atrophy (GA) growth without adverse events in participants with GA secondary to AMD when administered subcutaneously. (Jaffe et al., 2020; Liao et al., 2020)

Von Willebrand disease results from a defect in the vWF and is the most common inherited disorder causing mucocutaneous bleeding and excessive bleeding after trauma or invasive procedures. The vWF is responsible for regulating primary hemostasis and functions as a coagulation factor VIII carrier and (Calmette and Clauser, 2018) therefore, inhibition of the von Willebrand factor could offer a new approach to prevent excessive bleeding. ARC1779, a synthetically manufactured aptamer conjugated to a polyethylene glycol is an antagonist aptamer for the vWF A1 domain, a ligand for receptor glycoprotein 1b on platelets. The ARC1779 has shown well tolerability and dose- and concentration-dependent reduction in vWF activity with no bleeding. It also showed the ability to inhibit von Willebrand factor-dependent platelet function in patients with thrombotic thrombocytopenic purpura (NCT00632242) (Gilbert et al., 2007; Mayr et al., 2010).

Mirror-image single-stranded oligonucleotides called Spiegelmers have also been designed to target different proteins. These mirror-image aptamers contain L-stereoisomeric oligonucleotides that are stable due to their mirror-image conformation. Though these mirror‐image oligonucleotides exhibit the same physical and chemical properties as their d‐counterparts, they resist nuclease degradation and off‐target interactions. (Oberthür et al., 2015; Young et al., 2019) The first step of Spiegelmers synthesis involves the synthesis of the enantiomer. In the next step, a large collection of nucleic acids is screened. Using SELEX, an aptamer that binds to the non‐natural enantiomer is identified through 10–20 rounds of selection and the target is frequently biotinylated to isolate binding from nonbinding sequences using streptavidin- or neutravidin. Then the selected aptamer enantiomer is produced with a high affinity to their ligands. (Hornby, 2006; Vater and Klussmann, 2015) One of such pegylated L-ribonucleotide Spiegelmer^®^, NOX-H94 aptamer targeted hepcidin, a 25 amino acid peptide, and demonstrated a significant increase in Hb levels and increased red cell and reticulocyte hemoglobin in multiple myeloma and low-grade lymphoma patients. (Georgiev et al., 2014) Emapticap pegol (NOX-E36) is a 40-nucleotide oligonucleotide aptamer designed to target and inhibit C-C motif-ligand 2 (CCL2) (also called monocyte-chemotactic protein 1). In type 2 diabetic patients with albuminuria, subcutaneous injection of Emapticap showed well-tolerability and capability of inhibiting the CCL2/CCL2 receptor axis (Menne et al., 2017).

### Ribozyme

Ribozymes are catalytic RNA molecules that hybridize to target RNA and lead to the degradation of RNA. The result is that the fragmented RNA cannot participate in the translation thus inhibiting the production of a specific protein. Importantly, ribozymes can function without cellular proteins. (Sullivan, 1994) Therefore, it has attracted significant interest as a tool for gene manipulation. Their successful application in inhibiting gene expression not only *in vitro* but also *in vivo* has led to their clinical trials (Kashani-Sabet, 2002; Khan, 2006) for solid tumors, human immunodeficiency virus (HIV) and other diseases.

RPI.4610 (Angiozyme), a chemically stabilized anti-VEGFR-1 ribozyme in conjunction with carboplatin and paclitaxel in advanced solid tumor participants has shown to be safe (Kobayashi et al., 2005) with good bioavailability and localization ability in the tumor. (Weng et al., 2005) However, it failed demonstrate clinical efficacy in metastatic breast cancer patients and therefore was ruled out from further development (Morrow et al., 2012).

A tat-vpr-specific anti-HIV ribozyme, OZ1, (NCT00074997) delivered through autologous CD34^+^ cells showed a significant increase in CD4^+^ lymphocyte indicating that cell-delivered gene transfer is as much reliable in terms of maintaining safety and activeness of the ribozyme. (Mitsuyasu et al., 2009) Regardless of success in many clinical trials, few studies have also reported a lack of efficacy and safety, such as Ad5CRT targeting human telomerase reverse transcriptase (hTERT)-encoding RNAs in gastrointestinal cancer patients, angiozyme in metastatic breast cancer patients, or chimeric ribozyme. (Schiff et al., 2007; Morrow et al., 2012; Lee et al., 2019) Therefore, ribozymes still need improvement in terms of their stability, *in vivo* activeness, co-localization, delivery to the specific cell, as well as a for the continuation of stable and long-term expression (Khan, 2006).

### mRNA

The first report that shows the use of mRNA as nucleic acid-encoded drugs was observed over TWO decades ago when it was revealed that the administration of *in vitro* transcribed (IVT) mRNA could promote the expression of the encoded protein in the muscle, allowing mRNA manipulation to transitorily express proteins that have been suppressed in various diseases to resemble natural mRNA. (Sahin et al., 2014) When it comes to using mRNA as a therapeutic, it faces major problems such as immunity, delivery, and the ability to maintain potency. (Weissman, 2015) mRNA therapeutics have evolved into mRNA vaccines and protein replacement therapies. There is significant work involved to safely deliver mRNA into the cell while safeguarding the efficiency of mRNA and many such studies have made it into clinical trials including VEGF and cystic fibrosis transmembrane conductance regulator (CFTR) mRNA-based methods. (Trepotec et al., 2019a) The heightened innate immune response can be subdued by modification of 5′-untranslated region (5′-UTR) of mRNA. However, that can affect mRNA functionality. Nevertheless, it has been shown that minimizing 5′-UTR sequences can produce similar or higher expression than normal mRNA. (Trepotec et al., 2019b) The adverse symptoms of mRNA vaccine such as IFN-I responses can be generated possibly due to contamination of dsRNA. High-performance liquid chromatography (HPLC)-mediated removal can result in mRNA that does not activate innate immunity. (Karikó et al., 2011) A list of mRNA as a therapeutics in a clinical trial can be found in Table 3.

**TABLE 3:** mRNA in clinical trials.

Name	Disease	Target	ClinicalTrials.gov Identifier	Recruitment Status	Phase	Reference
mRNA-1273	COVID-19 vaccine	Spike (S) protein of SARS-CoV-2	NCT04283461	Active	I	Jackson et al. (2020)
BNT162b1	COVID-19 vaccine	RBD of Spike glycoprotein	NCT04368728	Recruiting	I	Mulligan et al. (2020)
mRNA-4157	Vaccine	Multiple neoantigens	NCT03313778	Recruiting	I	Burris et al. (2019)
Lipo-MERIT	Melanoma	Melanoma-associated antigens	NCT02410733	Active	I	(Jabulowsky et al., 2018; Sahin et al., 2020)
CV7201	Rabies	Rabies virus glycoprotein	NCT02241135	Completed	I	Alberer et al. (2017)
VAL-506440	Influenza	H10N8 Antigen	NCT03076385	Completed	I	Feldman et al. (2019)
VAL-339851		H7N9 antigen	NCT03345043	Active	I	
AZD8601	Type II Diabetes	VEGF-A	NCT02935712	Completed	I	Gan et al. (2019)
	Heart Failure		NCT03370887	Recruiting	II	Anttila et al. (2020)

#### mRNA as Vaccine

##### mRNA as Vaccines for Viral Diseases

Coronavirus disease 2019 (COVID-19) is caused by the severe acute respiratory syndrome coronavirus 2 (SARS-CoV-2) with a high fatality rate in older adults, especially those with comorbidities. (Vellas et al., 2020) Therefore, it is important to develop a vaccine that is safe and effective in preventing COVID-19. Currently, two mRNA vaccines are being used as preventive measures against COVID-19.

A vaccine candidate, mRNA-1273 contains a lipid nanoparticle–encapsulated, nucleoside-modified mRNA vaccine encoding the SARS-CoV-2 spike (S) glycoprotein stabilized in its prefusion conformation to enhance immune response. The study of mRNA-1273 in nonhuman primates has shown that the vaccine was able to stimulate antibody and T-cell responses. (Corbett et al., 2020) A dose-escalation study of mRNA-1273 in healthy adult humans showed a high antibody response with no trial-limiting safety concerns. (Jackson et al., 2020) BNT162b1 is another lipid-nanoparticle encapsulated, nucleoside-modified mRNA vaccine coding the trimerized receptor-binding domain (RBD) of the spike glycoprotein of SARS-CoV-2. The BNT162b1 expressed antigen contains an RBD modified by the addition of a T4 fibritin-derived foldon trimerization domain to increase its immunogenicity by the multivalent display. The second dose of BNT162b1 has shown significant immunogenicity and improved RBD-binding IgG concentrations and SARS-CoV-2 neutralizing titers (Mulligan et al., 2020) Both of these vaccines, mRNA-1273 and BNT162b1, have received emergency use authorization (EUA) from the U.S. Food and Drug Administration (USFDA). (USFDA, 2021a; USFDA, 2021b) mRNA vaccines for the Zika virus (ZIKV) have also been developed. These vaccines contained a modified mRNA encoding pre-membrane and envelope glycoproteins (wild-type or variant ZIKV structural gene) encased in a modified lipid nanoparticle. The resultant vaccine was able to stimulate the production of neutralizing antibody titers against ZIKV infection. (Richner et al., 2017) Moreover, to avoid cross-reactivity altered pre-membrane and envelope (prM-E) RNA was designed to lower the production of antibodies enhancing DENV infection in mice without affecting the protection against ZIKV. (Richner et al., 2017) The nucleoside-modified mRNA, coding pre-membrane and envelope glycoproteins for ZIKV, could also produce a strong and long-lasting neutralizing antibody and ZIKV-specific T helper response in mice as well as in non-human primates. It also protected mice and non-human primates from ZIKV after the ZIKV challenge, establishing the ability of the vaccine to protect against ZIKV (Pardi et al., 2017).

Rabies virus glycoprotein (CV7201) coding prophylactic mRNA vaccine has also demonstrated to stimulate the production of antibodies against a viral antigen in human beings without safety concerns and with good tolerability. (Alberer et al., 2017) In other studies, mRNA vaccines against H10N8 and H7N9 demonstrated a favorable safety and reactogenicity with stimulated humoral immune responses (Feldman et al., 2019).

##### mRNA as Vaccine for Cancer

Apart from mRNA as vaccines for viral diseases, they also have shown promising results as cancer vaccines. mRNA-4157 in solid tumor (melanoma, colon, and lung cancers) patients in conjunction with pembrolizumab was well-tolerated without dose-limiting toxicities and also induced clinical responses in combination with pembrolizumab and neoantigen-specific T cells. (Burris et al., 2019) The Lipo-MERIT, a liposomal RNA targeting tumor-associated antigens in humans, also demonstrated well-tolerability and generated robust CD4^+^ and CD8^+^ T cell responses against vaccine antigens (Jabulowsky et al., 2018; Sahin et al., 2020).

#### mRNA as Protein Replacement Therapies

Apart from being used as vaccines, mRNAs are also employed in protein replacement therapies. An earlier study has highlighted the important role of a 165-amino-acid isoform of VEGF-A (VEGF-A_165_) in the formation of new blood vessels and improving cardiac function post-myocardial infarction in swine. (Carlsson et al., 2018) AZD8601, a VEGF-A_165_ mRNA formulation, has been shown to stimulate the production of VEGF-A protein, enhancing basal skin blood flow in men with type 2 diabetes mellitus (T2DM). (Gan et al., 2019) In the follow-up study, AZD8601, in patients with obstructive coronary artery disease, is being evaluated for safety and angiogenic effects of VEGF-A mRNA on myocardial perfusion and cardiac function (Anttila et al., 2020).

### CRISPR/Cas9

CRISPR-CRISPR-associated protein 9 (Cas9) system is a bacterial defense mechanism that employs the guide RNA (gRNA) to mediate DNA endonuclease Cas9 to introduce site-specific breaks in target DNA. This ability of DNA editing has led CRISPR-Cas9 to be used as RNA-therapeutic in genome modification for biological and therapeutic applications by synthesizing single-guide RNA (sgRNA). (Ma et al., 2014; Jiang and Doudna, 2017) Many studies have shown the applications of CRISPR/Cas9 to treat genetic disorders such as cystic fibrosis (CF), Duchenne muscular dystrophy (DMD), and hemoglobinopathies as well as HIV and β-thalassemia. In CF, CRISPR/Cas9 has shown to correct cystic fibrosis transmembrane conductor receptor (CFTR) in cultured intestinal stem cells, restoring the expression and function of the corrected gene. (Schwank et al., 2013) In DMD, mutations in the dystrophin coding gene promote myofiber disintegration and muscle deterioration. In a mouse model CRISPR-Cas9 with paired guide RNAs flanking the mutated Dmd exon23 was successfully able to remove the mutated part and partially improve muscle function. (Tabebordbar et al., 2016) In another study, CRISPR/Cas9 was able to increase the utrophin amount and remove duplicated DMD exons 18–30 in myotubes, resulting in the production of full-length dystrophin in DMD. (Wojtal et al., 2016) In β-thalassemia, mutations in the human hemoglobin beta (HBB) gene can be corrected by CRISPR/Cas9. With piggyBac transposon, CRISPR/Cas9 in patient-derived iPSCs effectively corrected HBB mutations without affecting the pluripotency of iPSCs and restored the expression of HBB when differentiated into erythroblasts. (Xie et al., 2014) A similar application of CRISPR-Cas9 has also been demonstrated in immune disorders such as AIDS. The study has shown that HIV-1–directed guide RNAs in infected microglial, pro-monocytic, and T cells could target the HIV-1 long terminal repeats U3 region and excise a 9,709-bp integrated proviral DNA to repress viral gene expression and replication (Hu et al., 2014).

Although these studies provide the background for future CRISPR-Cas9 clinical trials, some challenges remain to be addressed before CRISPR/Cas9 can further be employed in clinical trials and subsequently in treatments. The particular challenges include the delivery of gene-editing tools to the target cells, exclusively *in vivo* and possible off-target effects. (Redman et al., 2016) In addition, ethical concerns and germline applications of CRISPR-Cas9 also need to be considered before translating CRISPR-Cas9 into therapeutic applications.

### Currently Approved RNA Therapeutics

Despite the numbers of RNA therapeutics in clinical trials for various diseases, only a few have been approved for public use by the USFDA. The list can be seen in Table 4. The first-ever RNA therapy that was approved by the USFDA is fomivirsen for the treatment of cytomegalovirus retinitis, a manifestation of DNA herpes cytomegalovirus (CMV), commonly seen in advanced acquired immunodeficiency syndrome (AIDS). (Port et al., 2017) siRNA-based therapeutics for hereditary transthyretin-mediated amyloidosis and acute hepatic porphyria (AHP) have also been approved by USFDA. Patisiran, a lipid nanoparticle formulation of siRNA has been designed to specifically inhibit transthyretin synthesis in the liver, (Adams et al., 2018; Hoy, 2018) while givosiran, a synthetic siRNA targeted towards 5-aminolevulinic acid synthase (ALAS1) is approved for the treatment of AHP. (USFDA, 2019) Pegaptanib, a 28-nucleotides RNA aptamer targeting VEGF_165_ approved in 2004 remains to be the only aptamer for AMD (Vinores, 2006).

**TABLE 4:** Clinically approved RNA therapeutics.

Drug	Molecule	Target	Disease	Reference
mRNA-1273	mRNA	Spike (S) protein of SARS-CoV-2	COVID-19	USFDA, (2021a)
BNT162b1		RBD of Spike glycoprotein		USFDA, (2021b)
Patisiran	siRNA	Polyneuropathy	Hereditary transthyretin-mediated amyloidosis	Adams et al. (2018)
Givosiran	siRNA	ALAS1	AHP	USFDA, (2019)
Pegaptanib	Aptamer	VEGF	AMD	Vinores, (2006)

## Delivery Systems for RNA Therapeutics

RNAs are single-stranded nucleic acid molecules and therefore, often targeted by nucleases and eliminated from the body. Therefore, to function *in vivo*, RNAs need to be protected and delivered to the intended site. This can be accomplished by different delivery systems. Recently viral vectors and non-viral vectors have been reported as carriers for effective and safe delivery of RNAs. (Patil et al., 2019) Such delivery systems include cationic lipids (1,2-dioleoyl-3-trimethylammonium-propane (DOTAP), dimethyl dioctadecyl ammonium bromide (DDAB), and cetyl-trimethylammonium bromide (CTAB)), quantum dots, metal complexes, and fluorescent nanoparticles, polymers such as chitosan, dendrimers, polyethylenimine (PEI), polyaminoester (PAE), polyacrylic acid (PAA), and polyamidoamine (PAMAM). These delivery systems are non-immunogenic, less toxic, but often have low transfection efficiency *in vivo* and therefore, require modifications. (Patil et al., 2019) For example, the addition of thiol groups to gelatin facilitates disulfide bond formation within the polymer strengthening the structure of protein and improving nanoparticle stability during systemic circulation. Such modified nanoparticles can effectively encapsulate nucleic acids and enhance transfection efficiency, while PEGylation increase the size of particles decreasing their clearance from the kidney, (Madkhali et al., 2019) or hydrazone bond and disulfide bond addition to PEI for effective oligonucleotide release (Hao et al., 2019) or alkyl-PEG modification of cholesterol-grafted PAMAM dendrimers to enhance transfection efficiency by helping to evade extracellular and intracellular barriers (Pishavar et al., 2018) or phenylboronic acid-functionalization to facilitate and protect miR-34a against nuclease degradation or chondroitin sulfate-functionalization to polyamidoamine through Michael addition for efficient cellular uptake and intracellular transfection of miR-34a for tumor therapy (Chen et al., 2017; Song et al., 2019) have been reported. Moreover, the addition of targeting moieties such as D-Asp8 to polyurethane or DOTAP could enhance selective miRNA delivery to bone cells (Liu et al., 2015; Sun et al., 2016; Cai et al., 2017).

## Chemical Modifications of RNA

It is equally important that RNAs used in therapeutics should also be modified to avoid nuclease degradation and recognition by the immune system. (Kaczmarek et al., 2017) The nucleic acids are not only required to be modified to avoid nuclease degradation or rapid renal filtration but also require additional modifications to bind target molecules (Flamme et al., 2019).

**FIGURE 1:**
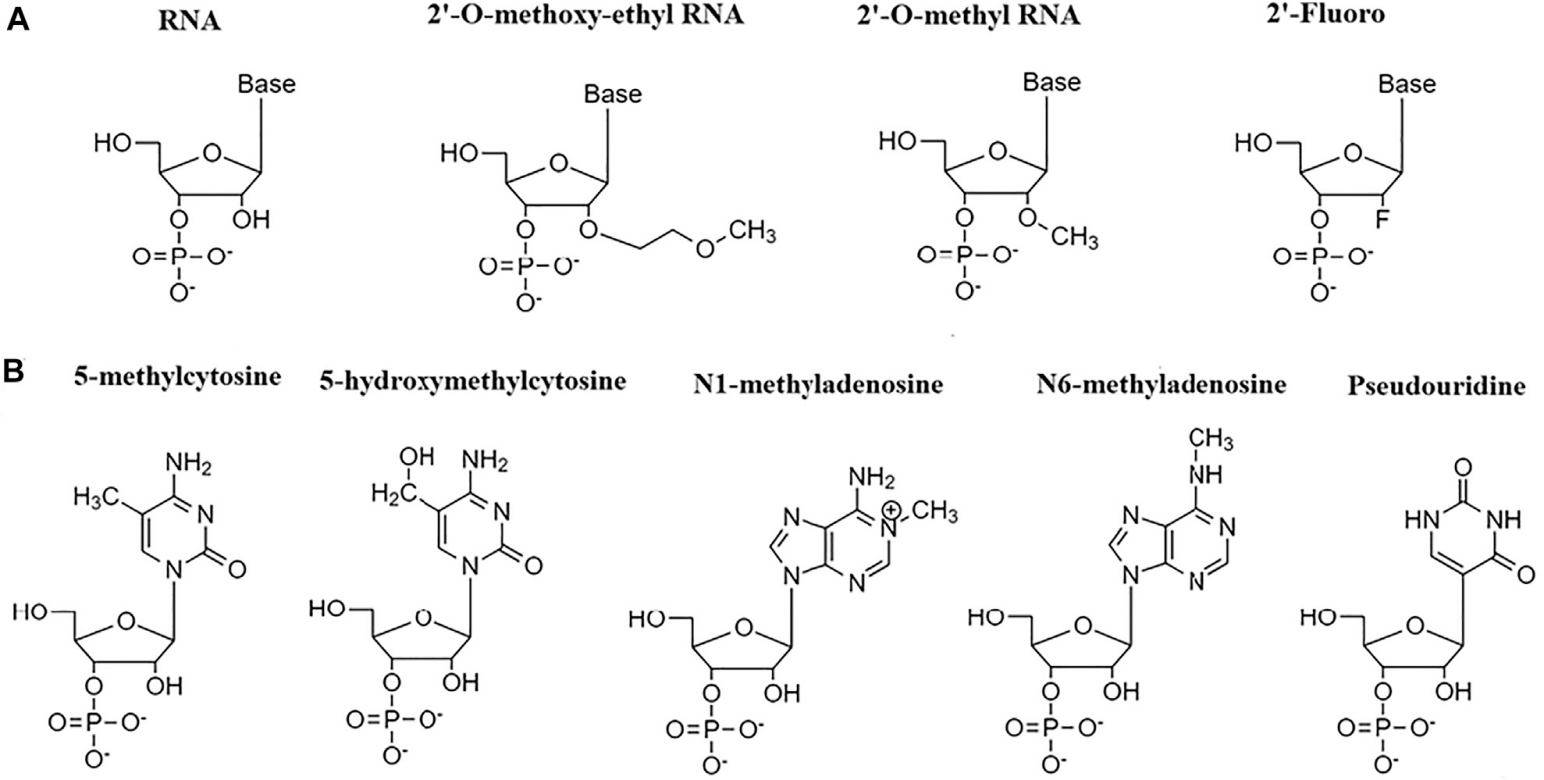
RNA modifications. **(A)** RNA modifications at 2′ position of the ribose sugar ring. **(B)** RNA modifications at nitrogen bases.

Many different RNA modifications, biological and chemical have been documented so far. RNA modifications, such as N6-methyladenosine (m^6^A), 5-methylcytosine (m^5^C), pseudouridine (Ψ), 5-hydroxymethylcytosine (hm^5^C), and N1-methyladenosine (m^1^A) have been demonstrated to regulate mRNA stability. (Song and Yi, 2017) (Huang et al., 2020) The coordinated actions of three proteins such as RNA-modifying enzymes called writer proteins (transfers chemical group to RNA molecules), RNA-binding proteins (RBPs) (recognize the altered nucleotide), and eraser proteins (removes the added chemical group from the modified nucleotide) determine the fate and stability of mRNA by modifying RNA. (Boo and Kim, 2020) The writer proteins that can carry m^6^A modification involve methyltransferase like 3 (METTL3), METTL14, WTAP, and KIAA1429, m5C modification involve NOP2/Sun RNA methyltransferase 2 (NSUN2), and pseudouridine modification involve pseudouridines synthases (PUSs), while RBPs that can carry m6A modification involve IGF2BPs, FMRP, G3BP1, PRRC2A, YT521-B homology (YTH) domain-containing proteins (YTHDF1, YTHDF2 YTHDF3, YTHDC2), and HuR, m5C modification involve Y-box binding protein 1 (YBX1) and m6A erasers such as α-ketoglutarate-dependent dioxygenase alkB homolog 5 protein (ALKBH5) remove the methyl group from m6A and converts it into adenosine. (Boo and Kim, 2020) The previous report has suggested that chemical siRNA modifications at 2′ position of ribose sugar ring such as 2′-O-methyl (2′-O-me), 2′-Fluoro (2′-F), 2′-O-methoxyethyl (2′-MOE). (Figure 1A) allow oligonucleotide to adopt RNA-like C3′-endo sugar pucker, making it thermally stable. (Selvam et al., 2017) The stability and resistance to serum is also shown to be improved without any significant RNA interference loss upon 2′-O-methyl modifications in siRNAs. (Czauderna et al., 2003) The addition of guanidinopropyl (GP) moiety at 2′ of sugar ring (2′-O-GP) also improved the silencing ability of modified siRNA in the hepatitis B virus (HBV). (Brzezinska et al., 2012) CircRNAs are often used in gene therapy. Despite their circular nature, circRNA can prompt an innate immune response. The study has shown that modifying RNA with N^6^-methyladenosine can suppress circRNA immunity by marking it as self RNA (Chen et al., 2019).

## Conclusion

In recent years, RNA-based therapeutics evolved into a potential possible intervention strategy in various diseases. Generally, RNA therapeutics are divided into different categories based on their mode of action and molecule used, such as siRNA-based, miRNA-based, antisense oligonucleotides, aptamers, and mRNA vaccines. Many RNA therapeutics have been developed for various diseases and promising results obtained in many clinical studies have led to their approval for the treatment of various diseases. However, the major hurdle in the development of RNA therapeutics in many diseases is the lack of suitable delivery systems. Therefore, much work is needed in the development of delivery systems with higher targeting ability and less toxicity (Markus et al., 2011; Doudna and Charpentier, 2014; Mehta, 2015; Li et al., 2018).

## References

[B1] Aagaard L. Rossi J. J. (2007). RNAi Therapeutics: Principles, Prospects and Challenges. Adv. Drug Deliv. Rev. 59 (2-3), 75–86. 10.1016/j.addr.2007.03.005 17449137 PMC1978219

[B2] Adachi T. Nakamura Y. (2019). Aptamers: A Review of Their Chemical Properties and Modifications for Therapeutic Application. Molecules 24 (23). 10.3390/molecules24234229 PMC6930564 31766318

[B3] Adams D. Gonzalez-Duarte A. O'Riordan W. D. Yang C. C. Ueda M. Kristen A. V. (2018). Patisiran, an RNAi Therapeutic, for Hereditary Transthyretin Amyloidosis. N. Engl. J. Med. 379 (1), 11–21. 10.1056/NEJMoa1716153 29972753

[B4] Alberer M. Gnad-Vogt U. Hong H. S. Mehr K. T. Backert L. Finak G. (2017). Safety and Immunogenicity of a mRNA Rabies Vaccine in Healthy Adults: An Open-Label, Non-randomised, Prospective, First-In-Human Phase 1 Clinical Trial. The Lancet 390 (10101), 1511–1520. 10.1016/s0140-6736(17)31665-3 28754494

[B5] Altamura S. Altamura S. Muckenthaler M. U. Dames S. Frauendorf C. Schubert S. (2018). SLN124, a Galnac-siRNA Conjugate Targeting TMPRSS6, for the Treatment of Iron Overload and Ineffective Erythropoiesis Such as in Beta-Thalassemia. Blood 132 (Suppl. 1), 2340. 10.1182/blood-2018-99-110163

[B6] Altamura S. Schaeper U. Dames S. Löffler K. Eisermann M. Frauendorf C. (2019). SLN124, a GalNAc-siRNA Conjugate Targeting TMPRSS6, Efficiently Prevents Iron Overload in Hereditary Haemochromatosis Type 1. HemaSphere 3 (6), e301. 10.1097/hs9.0000000000000301 31976476 PMC6924545

[B7] Amarzguioui M. Holen T. Babaie E. Prydz H. (2003). Tolerance for Mutations and Chemical Modifications in a siRNA. Nucleic Acids Res. 31 (2), 589–595. 10.1093/nar/gkg147 12527766 PMC140512

[B8] Amarzguioui M. Rossi J. J. Kim D. (2005). Approaches for Chemically Synthesized siRNA and Vector-Mediated RNAi. FEBS Lett. 579 (26), 5974–5981. 10.1016/j.febslet.2005.08.070 16199038

[B9] Andreoli C. M. Miller J. W. (2007). Anti-vascular Endothelial Growth Factor Therapy for Ocular Neovascular Disease. Curr. Opin. Ophthalmol. 18 (6), 502–508. 10.1097/icu.0b013e3282f0ca54 18163003

[B10] Antoszyk A. Katz B. Singh R. P. Gurses-Ozden R. Erlich S. Rothenstein D. (2013). A Phase I Open Label, Dose Escalation Trial of QPI-1007 Delivered by A Single Intravitreal (IVT) Injection to Subjects with Low Visual Acuity and Acute Non-arteritic Anterior Ischemic Optic Neuropathy (NAION). Invest. Ophthalmol. Vis. Sci. 54 (15), 4575.

[B11] Anttila V. Saraste A. Knuuti J. Jaakkola P. Hedman M. Svedlund S. (2020). Synthetic mRNA Encoding VEGF-A in Patients Undergoing Coronary Artery Bypass Grafting: Design of a Phase 2a Clinical Trial. Mol. Ther. - Methods Clin. Development 18, 464–472. 10.1016/j.omtm.2020.05.030 PMC7369517 32728595

[B12] Atri C. Guerfali F. Z. Laouini D. (2019). “MicroRNAs in Diagnosis and Therapeutics,” in AGO-driven Non-coding RNAs. Editor Mallick B. (Amsterdam, Netherlands: Academic Press), 137–177. 10.1016/b978-0-12-815669-8.00006-3

[B13] Banerjee J. Nilsen-Hamilton M. (2013). Aptamers: Multifunctional Molecules for Biomedical Research. J. Mol. Med. 91 (12), 1333–1342. 10.1007/s00109-013-1085-2 24045702

[B14] Bekris L. M. Leverenz J. B. (2015). The Biomarker and Therapeutic Potential of miRNA in Alzheimer's Disease. Neurodegenerative Dis. Management 5 (1), 61–74. 10.2217/nmt.14.52 25711455

[B15] Boo S. H. Kim Y. K. (2020). The Emerging Role of RNA Modifications in the Regulation of mRNA Stability. Exp. Mol. Med. 52 (3), 400–408. 10.1038/s12276-020-0407-z 32210357 PMC7156397

[B16] Braasch D. A. Jensen S. Liu Y. Kaur K. Arar K. White M. A. (2003). RNA Interference in Mammalian Cells by Chemically-Modified RNA. Biochemistry 42 (26), 7967–7975. 10.1021/bi0343774 12834349

[B17] Brzezinska J. D’Onofrio J. Buff M. C. R. Hean J. Ely A. Marimani M. (2012). Synthesis of 2′-O-Guanidinopropyl-Modified Nucleoside Phosphoramidites and Their Incorporation into siRNAs Targeting Hepatitis B Virus. Bioorg. Med. Chem. 20 (4), 1594–1606. 10.1016/j.bmc.2011.12.024 22264759

[B18] Burris H. A. Patel M. R. Cho D. C. Clarke J. M. Gutierrez M. Zaks T. Z. (2019). A Phase I Multicenter Study to Assess the Safety, Tolerability, and Immunogenicity of mRNA-4157 Alone in Patients with Resected Solid Tumors and in Combination with Pembrolizumab in Patients with Unresectable Solid Tumors. Jco 37 (15_Suppl. l), 2523. 10.1200/jco.2019.37.15_suppl.2523

[B19] Cai M. Yang L. Zhang S. Liu J. Sun Y. Wang X. (2017). A Bone-Resorption Surface-Targeting Nanoparticle to Deliver Anti-miR214 for Osteoporosis Therapy. Ijn 12, 7469–7482. 10.2147/ijn.s139775 29075114 PMC5648312

[B20] Calmette L. Clauser S. (2018). La maladie de Willebrand. La Revue de Médecine Interne 39 (12), 918–924. 10.1016/j.revmed.2018.08.005 30279008

[B21] Carlsson L. Clarke J. C. Yen C. Gregoire F. Albery T. Billger M. (2018). Biocompatible, Purified VEGF-A mRNA Improves Cardiac Function After Intracardiac Injection 1 Week Post-myocardial Infarction in Swine. Mol. Ther. - Methods Clin. Development 9, 330–346. 10.1016/j.omtm.2018.04.003 PMC6054703 30038937

[B22] Chen W. Liu Y. Liang X. Huang Y. Li Q. (2017). Chondroitin Sulfate-Functionalized Polyamidoamine as a Tumor-Targeted Carrier for miR-34a Delivery. Acta Biomater. 57, 238–250. 10.1016/j.actbio.2017.05.030 28511876

[B23] Chen Y. G. Chen R. Ahmad S. Verma R. Kasturi S. P. Amaya L. (2019). N6-Methyladenosine Modification Controls Circular RNA Immunity. Mol. Cel 76 (1), 96–109.e9. 10.1016/j.molcel.2019.07.016 PMC6778039 31474572

[B24] Chi X. Gatti P. Papoian T. (2017). Safety of Antisense Oligonucleotide and siRNA-Based Therapeutics. Drug Discov. Today 22 (5), 823–833. 10.1016/j.drudis.2017.01.013 28159625

[B25] Ciccarelli G. D'Elia S. De Paulis M. Golino P. Cimmino G. (2018). Lipid Target in Very High-Risk Cardiovascular Patients: Lesson from PCSK9 Monoclonal Antibodies. Diseases 6 (1), 22. 10.3390/diseases6010022 PMC5871968 29562587

[B26] Corbett K. S. Flynn B. Foulds K. E. Francica J. R. Boyoglu-Barnum S. Werner A. P. (2020). Evaluation of the mRNA-1273 Vaccine against SARS-CoV-2 in Nonhuman Primates. New Engl. J. Med. 383 (16), 1544–1555. 10.1056/NEJMoa2024671 32722908 PMC7449230

[B27] Czauderna F. Fechtner M. Dames S. Aygün H. Klippel A. Pronk G. J. (2003). Structural Variations and Stabilising Modifications of Synthetic siRNAs in Mammalian Cells. Nucleic Acids Res. 31 (11), 2705–2716. 10.1093/nar/gkg393 12771196 PMC156727

[B28] Davis M. E. Zuckerman J. E. Choi C. H. J. Seligson D. Tolcher A. Alabi C. A. (2010). Evidence of RNAi in Humans from Systemically Administered siRNA via Targeted Nanoparticles. Nature 464 (7291), 1067–1070. 10.1038/nature08956 20305636 PMC2855406

[B29] De Majo F. De Windt L. J. (2018). RNA Therapeutics for Heart Disease. Biochem. Pharmacol. 155, 468–478. 10.1016/j.bcp.2018.07.037 30059676

[B30] Demeure M. J. Armaghany T. Ejadi S. Ramanathan R. K. Elfiky A. Strosberg J. R. (2016). A Phase I/II Study of TKM-080301, a PLK1-Targeted RNAi in Patients with Adrenocortical Cancer (ACC). Jco 34 (15_Suppl. l), 2547. 10.1200/jco.2016.34.15_suppl.2547

[B31] Demirjian S. Ailawadi G. Polinsky M. Bitran D. Silberman S. Shernan S. K. (2017). Safety and Tolerability Study of an Intravenously Administered Small Interfering Ribonucleic Acid (siRNA) Post On-Pump Cardiothoracic Surgery in Patients at Risk of Acute Kidney Injury. Kidney Int. Rep. 2 (5), 836–843. 10.1016/j.ekir.2017.03.016 29270490 PMC5733816

[B32] den Hollander A. I. Roepman R. Koenekoop R. K. Cremers F. P. M. (2008). Leber Congenital Amaurosis: Genes, Proteins and Disease Mechanisms. Prog. Retin. Eye Res. 27 (4), 391–419. 10.1016/j.preteyeres.2008.05.003 18632300

[B33] Martin D. F. Klein M. Haller J. Adamis A. Miller J. Blumenkrantz M. (2002). Preclinical and Phase 1A Clinical Evaluation of an Anti-VEGF Pegylated Aptamer (EYE001) for the Treatment of Exudative Age-Related Macular Degeneration. Retina 22 (2), 143–152. 10.1097/00006982-200204000-00002 11927845

[B34] Do H. D. Vandermies M. Fickers P. Theron C. W. (2019). “Non-Conventional Yeast Species for Recombinant Protein and Metabolite Production,” in Reference Module in Life Sciences (Elsevier). 10.1016/b978-0-12-809633-8.20885-6

[B35] Doudna J. A. Charpentier E. (2014). The New Frontier of Genome Engineering with CRISPR-Cas9. Science 346 (6213), 1258096. 10.1126/science.1258096 25430774

[B36] Dulla K. Aguila M. Lane A. Jovanovic K. Parfitt D. A. Schulkens I. (2018). Splice-Modulating Oligonucleotide QR-110 Restores CEP290 mRNA and Function in Human c.2991+1655A>G LCA10 Models. Mol. Ther. - Nucleic Acids 12, 730–740. 10.1016/j.omtn.2018.07.010 30114557 PMC6092551

[B37] Dutta C. Avitahl-Curtis N. Pursell N. Larsson Cohen M. Holmes B. Diwanji R. (2016). Inhibition of Glycolate Oxidase with Dicer-Substrate siRNA Reduces Calcium Oxalate Deposition in a Mouse Model of Primary Hyperoxaluria Type 1. Mol. Ther. 24 (4), 770–778. 10.1038/mt.2016.4 26758691 PMC4886950

[B38] El Dika I. Lim H. Y. Yong W. P. Lin C. C. Yoon J. H. Modiano M. (2019). An Open‐Label, Multicenter, Phase I, Dose Escalation Study with Phase II Expansion Cohort to Determine the Safety, Pharmacokinetics, and Preliminary Antitumor Activity of Intravenous TKM‐080301 in Subjects with Advanced Hepatocellular Carcinoma. Oncol. 24 (6), 747-e218. 10.1634/theoncologist.2018-0838 PMC6656521 30598500

[B40] Feldman R. A. Fuhr R. Smolenov I. Ribeiro A. Panther L. Watson M. (2019). mRNA Vaccines against H10N8 and H7N9 Influenza Viruses of Pandemic Potential Are Immunogenic and Well Tolerated in Healthy Adults in Phase 1 Randomized Clinical Trials. Vaccine 37 (25), 3326–3334. 10.1016/j.vaccine.2019.04.074 31079849

[B41] Flamme M. McKenzie L. K. Sarac I. Hollenstein M. (2019). Chemical Methods for the Modification of RNA. Methods 161, 64–82. 10.1016/j.ymeth.2019.03.018 30905751

[B42] Gan L.-M. Lagerström-Fermér M. Carlsson L. G. Arfvidsson C. Egnell A.-C. Rudvik A. (2019). Intradermal Delivery of Modified mRNA Encoding VEGF-A in Patients with Type 2 Diabetes. Nat. Commun. 10 (1), 871. 10.1038/s41467-019-08852-4 30787295 PMC6382754

[B43] Gao Y. Patil S. Qian A. (2020). The Role of MicroRNAs in Bone Metabolism and Disease. Ijms 21 (17), 6081. 10.3390/ijms21176081 PMC7503277 32846921

[B44] Gavrilov K. Saltzman W. M. (2012). Therapeutic siRNA: Principles, Challenges, and Strategies. Yale J. Biol. Med. 85 (2), 187–200. 22737048 PMC3375670

[B45] Georgiev P. Lazaroiu M. Ocroteala L. Grudeva-Popova J. Gheorghita E. Vasilica M. (2014). Abstract 3847: The Anti-hepcidin Spiegelmer® Lexaptepid Pegol (NOX-H94) as Treatment of Anemia of Chronic Disease in Patients with Multiple Myeloma, Low Grade Lymphoma, and CLL: A Phase II Pilot Study. Cancer Res. 74 (19), 3847. 10.1158/1538-7445.AM2014-3847

[B46] Gilbert J. C. DeFeo-Fraulini T. Hutabarat R. M. Horvath C. J. Merlino P. G. Marsh H. N. (2007). First-in-Human Evaluation of Anti-von Willebrand Factor Therapeutic Aptamer ARC1779 in Healthy Volunteers. Circulation 116 (23), 2678–2686. 10.1161/circulationaha.107.724864 18025536

[B47] Golan T. Khvalevsky E. Z. Hubert A. Gabai R. M. Hen N. Segal A. (2015). RNAi Therapy Targeting KRAS in Combination with Chemotherapy for Locally Advanced Pancreatic Cancer Patients. Oncotarget 6 (27), 24560–24570. 10.18632/oncotarget.4183 26009994 PMC4695206

[B48] Goroshchuk O. Kolosenko I. Vidarsdottir L. Azimi A. Palm-Apergi C. (2019). Polo-like Kinases and Acute Leukemia. Oncogene 38 (1), 1–16. 10.1038/s41388-018-0443-5 30104712

[B49] Hao F. Li Y. Zhu J. Sun J. Marshall B. Lee R. J. (2019). Polyethylenimine-based Formulations for Delivery of Oligonucleotides. Cmc 26 (13), 2264–2284. 10.2174/0929867325666181031094759 30378483

[B50] Hattori Y. Kikuchi T. Nakamura M. Ozaki K. I. Onishi H. (2017). Therapeutic Effects of Protein Kinase N3 Small Interfering RNA and Doxorubicin Combination Therapy on Liver and Lung Metastases. Oncol. Lett. 14 (5), 5157–5166. 10.3892/ol.2017.6830 29098022 PMC5652245

[B51] Hornby P. J. (2006). Designing Spiegelmers to Antagonise Ghrelin. Gut 55 (6), 754–755. 10.1136/gut.2005.076067 16698747 PMC1856213

[B52] Hoy S. M. (2018). Patisiran: First Global Approval. Drugs 78 (15), 1625–1631. 10.1007/s40265-018-0983-6 30251172

[B53] Hu W. Kaminski R. Yang F. Zhang Y. Cosentino L. Li F. (2014). RNA-directed Gene Editing Specifically Eradicates Latent and Prevents New HIV-1 Infection. Proc. Natl. Acad. Sci. 111 (31), 11461–11466. 10.1073/pnas.1405186111 25049410 PMC4128125

[B54] Huang H. Weng H. Chen J. (2020). m6A Modification in Coding and Non-coding RNAs: Roles and Therapeutic Implications in Cancer. Cancer Cell 37 (3), 270–288. 10.1016/j.ccell.2020.02.004 32183948 PMC7141420

[B55] Ill C. R. Chiou H. C. (2005). Gene Therapy Progress and Prospects: Recent Progress in Transgene and RNAi Expression Cassettes. Gene Ther. 12 (10), 795–802. 10.1038/sj.gt.3302524 15815698

[B56] Jabulowsky R. A. Loquai C. Mitzel-Rink H. Utikal J. Gebhardt C. Hassel J. C. (2018). Abstract CT156: A First-In-Human Phase I/II Clinical Trial Assessing Novel mRNA-Lipoplex Nanoparticles Encoding Shared Tumor Antigens for Immunotherapy of Malignant Melanoma. Cancer Res. 78 (13), CT156. 10.1158/1538-7445.am2018-ct156

[B57] Jackson L. A. Anderson E. J. Rouphael N. G. Roberts P. C. Makhene M. Coler R. N (2020). An mRNA Vaccine against SARS-CoV-2 — Preliminary Report. New Engl. J. Med. 383, 1920-1931. 10.1056/NEJMoa2022483 32663912 PMC7377258

[B58] Jaffe G. J. Ciulla T. A. Ciardella A. P. Devin F. Dugel P. U. Eandi C. M. (2017). Dual Antagonism of PDGF and VEGF in Neovascular Age-Related Macular Degeneration. Ophthalmology 124 (2), 224–234. 10.1016/j.ophtha.2016.10.010 28029445

[B59] Jaffe G. J. Eliott D. Wells J. A. Prenner J. L. Papp A. Patel S. (2016). A Phase 1 Study of Intravitreous E10030 in Combination with Ranibizumab in Neovascular Age-Related Macular Degeneration. Ophthalmology 123 (1), 78–85. 10.1016/j.ophtha.2015.09.004 26499921

[B60] Jaffe G. J. Westby K. Csaky K. G. Monés J. Pearlman J. A. Patel S. S. (2020). C5 Inhibitor Avacincaptad Pegol for Geographic Atrophy Due to Age-Related Macular Degeneration: A Randomized Pivotal Phase 2/3 Trial. Ophthalmology 128 (1), 576–586. 10.1016/j.ophtha.2020.08.027 32882310

[B61] Jiang F. Doudna J. A. (2017). CRISPR-Cas9 Structures and Mechanisms. Annu. Rev. Biophys. 46, 505–529. 10.1146/annurev-biophys-062215-010822 28375731

[B62] Kaczmarek J. C. Kowalski P. S. Anderson D. G. (2017). Advances in the Delivery of RNA Therapeutics: From Concept to Clinical Reality. Genome Med. 9 (1), 60. 10.1186/s13073-017-0450-0 28655327 PMC5485616

[B63] Kaiser P. K. Symons R. C. A. Shah S. M. Quinlan E. J. Tabandeh H. Do D. V. (2010). RNAi-based Treatment for Neovascular Age-Related Macular Degeneration by Sirna-027. Am. J. Ophthalmol. 150 (1), 33–39. 10.1016/j.ajo.2010.02.006 20609706

[B64] Karikó K. Muramatsu H. Ludwig J. Weissman D. (2011). Generating the Optimal mRNA for Therapy: HPLC Purification Eliminates Immune Activation and Improves Translation of Nucleoside-Modified, Protein-Encoding mRNA. Nucleic Acids Res. 39 (21), e142. 10.1093/nar/gkr695 21890902 PMC3241667

[B65] Kashani-Sabet M. (2002). Ribozyme Therapeutics. J. Invest. Dermatol. Symp. Proc. 7 (1), 76–78. 10.1046/j.1523-1747.2002.19642.x 12518796

[B66] Khan A. U. (2006). Ribozyme: A Clinical Tool. Clin. Chim. Acta 367 (1-2), 20–27. 10.1016/j.cca.2005.11.023 16426595

[B67] Kobayashi H. Gail Eckhardt S. Lockridge J. A. Rothenberg M. L. Sandler A. B. O’Bryant C. L. (2005). Safety and Pharmacokinetic Study of RPI.4610 (ANGIOZYME), an Anti-VEGFR-1 Ribozyme, in Combination with Carboplatin and Paclitaxel in Patients with Advanced Solid Tumors. Cancer Chemother. Pharmacol. 56 (4), 329–336. 10.1007/s00280-004-0968-x 15906031

[B68] Lander E. S. Linton L. M. Birren B. Nusbaum C. Zody M. C. Baldwin J. (2001). Initial Sequencing and Analysis of the Human Genome. Nature 409 (6822), 860–921. 10.1038/35057062 11237011

[B69] Leachman S. A. Hickerson R. P. Schwartz M. E. Bullough E. E. Hutcherson S. L. Boucher K. M. (2010). First-in-human Mutation-Targeted siRNA Phase Ib Trial of an Inherited Skin Disorder. Mol. Ther. 18 (2), 442–446. 10.1038/mt.2009.273 19935778 PMC2839285

[B70] Lee S. J. Shin S. P. Lee S. H. Kang J. W. Kook M. C. Kim I. H. (2019). Phase I Trial of Intravenous Ad5CRT in Patients with Liver Metastasis of Gastrointestinal Cancers. Cancer Gene Ther. 26 (5-6), 174–178. 10.1038/s41417-018-0055-9 30393375

[B71] Lei B. Tian Z. Fan W. Ni B. (2019). Circular RNA: A Novel Biomarker and Therapeutic Target for Human Cancers. Int. J. Med. Sci. 16 (2), 292–301. 10.7150/ijms.28047 30745810 PMC6367529

[B72] Lekka E. Hall J. (2018). Noncoding RNA S in Disease. FEBS Lett. 592 (17), 2884–2900. 10.1002/1873-3468.13182 29972883 PMC6174949

[B73] Li L. Hu S. Chen X. (2018). Non-viral Delivery Systems for CRISPR/Cas9-based Genome Editing: Challenges and Opportunities. Biomaterials 171, 207–218. 10.1016/j.biomaterials.2018.04.031 29704747 PMC5944364

[B74] Liao D. S. Grossi F. V. El Mehdi D. Gerber M. R. Brown D. M. Heier J. S. (2020). Complement C3 Inhibitor Pegcetacoplan for Geographic Atrophy Secondary to Age-Related Macular Degeneration. Ophthalmology 127 (2), 186–195. 10.1016/j.ophtha.2019.07.011 31474439

[B75] Liu J. Dang L. Li D. Liang C. He X. Wu H. (2015). A Delivery System Specifically Approaching Bone Resorption Surfaces to Facilitate Therapeutic Modulation of microRNAs in Osteoclasts. Biomaterials 52, 148–160. 10.1016/j.biomaterials.2015.02.007 25818421

[B76] Ma Y. Zhang L. Huang X. (2014). Genome Modification by CRISPR/Cas9. Febs j 281 (23), 5186–5193. 10.1111/febs.13110 25315507

[B77] Madkhali O. Mekhail G. Wettig S. D. (2019). Modified Gelatin Nanoparticles for Gene Delivery. Int. J. Pharmaceutics 554, 224–234. 10.1016/j.ijpharm.2018.11.001 30408531

[B78] Marín-garcía J. (2007). “Cardiovascular Gene Expression,” in Post-genomic Cardiology. Editor MarÍN-GarcÍA J. (Burlington, MA: Academic Press), 27–50. 10.1016/b978-012373698-7/50003-6

[B79] Markus H. S. McCollum C. Imray C. Goulder M. A. Gilbert J. King A. (2011). The von Willebrand Inhibitor ARC1779 Reduces Cerebral Embolization After Carotid Endarterectomy. Stroke 42 (8), 2149–2153. 10.1161/strokeaha.111.616649 21700934

[B80] Mayr F. B. Knöbl P. Jilma B. Siller-Matula J. M. Wagner P. G. Schaub R. G. (2010). The Aptamer ARC1779 Blocks Von Willebrand Factor-dependent Platelet Function in Patients with Thrombotic Thrombocytopenic Purpura Ex Vivo. Transfusion 50 (5), 1079–1087. 10.1111/j.1537-2995.2009.02554.x 20070617

[B81] Mehta S. (2015). Age-Related Macular Degeneration. Prim. Care Clin. Off. Pract. 42 (3), 377–391. 10.1016/j.pop.2015.05.009 26319344

[B82] Menne J. Eulberg D. Beyer D. Baumann M. Saudek F. Valkusz Z. (2017). C-C Motif-Ligand 2 Inhibition with Emapticap Pegol (NOX-E36) in Type 2 Diabetic Patients with Albuminuria. Nephrol. Dial. Transpl. 32 (2), 307–315. 10.1093/ndt/gfv459 PMC5410979 28186566

[B83] Milliner D. S. (2016). siRNA Therapeutics for Primary Hyperoxaluria: A Beginning. Mol. Ther. 24 (4), 666–667. 10.1038/mt.2016.50 27081720 PMC4886951

[B84] Mitchell P. Liew G. Gopinath B. Wong T. Y. (2018). Age-related Macular Degeneration. The Lancet 392 (10153), 1147–1159. 10.1016/s0140-6736(18)31550-2 30303083

[B85] Mitsuyasu R. T. Merigan T. C. Carr A. Zack J. A. Winters M. A. Workman C. (2009). Phase 2 Gene Therapy Trial of an Anti-HIV Ribozyme in Autologous CD34+ Cells. Nat. Med. 15 (3), 285–292. 10.1038/nm.1932 19219022 PMC2768566

[B86] Morikawa T. Hino R. Uozaki H. Maeda D. Ushiku T. Shinozaki A. (2010). Expression of Ribonucleotide Reductase M2 Subunit in Gastric Cancer and Effects of RRM2 Inhibition In Vitro. Hum. Pathol. 41 (12), 1742–1748. 10.1016/j.humpath.2010.06.001 20825972

[B87] Morrow P. K. Murthy R. K. Ensor J. D. Gordon G. S. Margolin K. A. Elias A. D. (2012). An Open-Label, Phase 2 Trial of RPI.4610 (Angiozyme) in the Treatment of Metastatic Breast Cancer. Cancer 118 (17), 4098–4104. 10.1002/cncr.26730 22281842

[B88] Moss K. H. Popova P. Hadrup S. R. Astakhova K. Taskova M. (2019). Lipid Nanoparticles for Delivery of Therapeutic RNA Oligonucleotides. Mol. Pharmaceutics 16 (6), 2265–2277. 10.1021/acs.molpharmaceut.8b01290 31063396

[B89] Mulligan M. J. Lyke K. E. Kitchin N. Absalon J. Gurtman A. Lockhart S. (2020). Phase I/II Study of COVID-19 RNA Vaccine BNT162b1 in Adults. Nature 586 (7830), 589–593. 10.1038/s41586-020-2639-4 32785213

[B90] Nguyen Q. D. Schachar R. A. Nduaka C. I. Sperling M. Basile A. S. Klamerus K. J. (2012). Dose-ranging Evaluation of Intravitreal siRNA PF-04523655 for Diabetic Macular Edema (The DEGAS Study). Invest. Ophthalmol. Vis. Sci. 53 (12), 7666–7674. 10.1167/iovs.12-9961 23074206

[B91] Nguyen Q. D. Schachar R. A. Nduaka C. I. Sperling M. Klamerus K. J. Chi-Burris K. (2012). Evaluation of the siRNA PF-04523655 Versus Ranibizumab for the Treatment of Neovascular Age-Related Macular Degeneration (MONET Study). Ophthalmology 119 (9), 1867–1873. 10.1016/j.ophtha.2012.03.043 22683252

[B92] Nimjee S. M. White R. R. Becker R. C. Sullenger B. A. (2017). Aptamers as Therapeutics. Annu. Rev. Pharmacol. Toxicol. 57, 61–79. 10.1146/annurev-pharmtox-010716-104558 28061688 PMC6035745

[B93] Oberthür D. Achenbach J. Gabdulkhakov A. Buchner K. Maasch C. Falke S. (2015). Crystal Structure of a Mirror-Image L-RNA Aptamer (Spiegelmer) in Complex with the Natural L-Protein Target CCL2. Nat. Commun. 6 (1), 6923. 10.1038/ncomms7923 25901662 PMC4423205

[B94] Pardi N. Hogan M. J. Pelc R. S. Muramatsu H. Andersen H. DeMaso C. R. (2017). Zika Virus Protection by a Single Low-Dose Nucleoside-Modified mRNA Vaccination. Nature 543 (7644), 248–251. 10.1038/nature21428 28151488 PMC5344708

[B95] Patil S. Gao Y. G. Lin X. Li Y. Dang K. Tian Y. (2019). The Development of Functional Non-viral Vectors for Gene Delivery. Int. J. Mol. Sci. 20 (21). 10.3390/ijms20215491 PMC6862238 31690044

[B96] Peddi V. Ratner L. Cooper M. Gaber O. Feng S. Tso P. (2014). Treatment with QPI-1002, a Short Interfering (SI) RNA for the Prophylaxis of Delayed Graft Function. Transplantation 98, 153.

[B97] Pishavar E. Attaranzadeh A. Alibolandi M. Ramezani M. Hashemi M. (2018). Modified PAMAM Vehicles for Effective TRAIL Gene Delivery to colon Adenocarcinoma: In Vitro and In Vivo Evaluation. Artif. Cell Nanomedicine, Biotechnol. 46 (Suppl. 3), S503–s513. 10.1080/21691401.2018.1500372 30095012

[B98] Ponzielli R. Katz S. Barsyte-Lovejoy D. Penn L. Z. (2005). Cancer Therapeutics: Targeting the Dark Side of Myc. Eur. J. Cancer 41 (16), 2485–2501. 10.1016/j.ejca.2005.08.017 16243519

[B99] Port A. D. Orlin A. Kiss S. Patel S. D'Amico D. J. Gupta M. P. (2017). Cytomegalovirus Retinitis: A Review. J. Ocul. Pharmacol. Ther. 33 (4), 224–234. 10.1089/jop.2016.0140 28355091

[B100] Raal F. J. Kallend D. Ray K. K. Turner T. Koenig W. Wright R. S. (2020). Inclisiran for the Treatment of Heterozygous Familial Hypercholesterolemia. N. Engl. J. Med. 382 (16), 1520–1530. 10.1056/nejmoa1913805 32197277

[B101] Ray K. K. Landmesser U. Leiter L. A. Kallend D. Dufour R. Karakas M. (2017). Inclisiran in Patients at High Cardiovascular Risk with Elevated LDL Cholesterol. N. Engl. J. Med. 376 (15), 1430–1440. 10.1056/nejmoa1615758 28306389

[B102] Reddy L. V. Miller T. M. (2015). RNA-targeted Therapeutics for ALS. Neurotherapeutics 12 (2), 424–427. 10.1007/s13311-015-0344-z 25753730 PMC4404448

[B103] Redman M. King A. Watson C. King D. (2016). What Is CRISPR/Cas9?. Arch. Dis. Child. Educ. Pract. Ed. 101 (4), 213–215. 10.1136/archdischild-2016-310459 27059283 PMC4975809

[B104] Richner J. M. Himansu S. Dowd K. A. Butler S. L. Salazar V. Fox J. M. (2017). Modified mRNA Vaccines Protect Against Zika Virus Infection. Cell 168 (6), 1114–1125. 10.1016/j.cell.2017.02.017 28222903 PMC5388441

[B105] Sahin U. Karikó K. Türeci Ö. (2014). mRNA-based Therapeutics - Developing A New Class of Drugs. Nat. Rev. Drug Discov. 13 (10), 759–780. 10.1038/nrd4278 25233993

[B106] Sahin U. Oehm P. Derhovanessian E. Jabulowsky R. A. Vormehr M. Gold M. (2020). An RNA Vaccine Drives Immunity in Checkpoint-Inhibitor-Treated Melanoma. Nature 585 (7823), 107–112. 10.1038/s41586-020-2537-9 32728218

[B107] Saw P. E. Song E.-W. (2020). siRNA Therapeutics: A Clinical Reality. Sci. China Life Sci. 63 (4), 485–500. 10.1007/s11427-018-9438-y 31054052

[B108] Schiff W. M. Hwang J. C. Ober M. D. Olson J. L. Dhrami-Gavazi E. Barile G. R. (2007). Safety and Efficacy Assessment of Chimeric Ribozyme to Proliferating Cell Nuclear Antigen to Prevent Recurrence of Proliferative Vitreoretinopathy. Arch. Ophthalmol. 125 (9), 1161–1167. 10.1001/archopht.125.9.1161 17846353

[B109] Schluep T. Lickliter J. Hamilton J. Lewis D. L. Lai C.-L. Lau J. Y. (2017). Safety, Tolerability, and Pharmacokinetics of ARC-520 Injection, an RNA Interference-Based Therapeutic for the Treatment of Chronic Hepatitis B Virus Infection, in Healthy Volunteers. Clin. Pharmacol. Drug Development 6 (4), 350–362. 10.1002/cpdd.318 PMC5516171 27739230

[B110] Schultheis B. Strumberg D. Kuhlmann J. Wolf M. Link K. Seufferlein T. (2016). A Phase Ib/IIa Study of Combination Therapy with Gemcitabine and Atu027 in Patients with Locally Advanced or Metastatic Pancreatic Adenocarcinoma. Jco 34 (4_Suppl. l), 385. 10.1200/jco.2016.34.4_suppl.385

[B111] Schultheis B. Strumberg D. Santel A. Vank C. Gebhardt F. Keil O. (2014). First-in-human Phase I Study of the Liposomal RNA Interference Therapeutic Atu027 in Patients with Advanced Solid Tumors. Jco 32 (36), 4141–4148. 10.1200/jco.2013.55.0376 25403217

[B112] Schwank G. Koo B.-K. Sasselli V. Dekkers J. F. Heo I. Demircan T. (2013). Functional Repair of CFTR by CRISPR/Cas9 in Intestinal Stem Cell Organoids of Cystic Fibrosis Patients. Cell Stem Cell 13 (6), 653–658. 10.1016/j.stem.2013.11.002 24315439

[B113] Scoles D. R. Minikel E. V. Pulst S. M. (2019). Antisense Oligonucleotides. Neurol. Genet. 5 (2), e323. 10.1212/nxg.0000000000000323 31119194 PMC6501637

[B114] Selvam C. Mutisya D. Prakash S. Ranganna K. Thilagavathi R. (2017). Therapeutic Potential of Chemically Modified siRNA: Recent Trends. Chem. Biol. Drug Des. 90 (5), 665–678. 10.1111/cbdd.12993 28378934 PMC5935465

[B115] Sivaprasad S. Chong N. V. (2006). The Complement System and Age-Related Macular Degeneration. Eye 20 (8), 867–872. 10.1038/sj.eye.6702176 16410816

[B116] Smith C. I. E. Blomberg P. (2017). [Gene Therapy – from Idea to Reality]. Lakartidningen 114, EWYL. 29297925

[B117] Solano E. C. R. Kornbrust D. J. Beaudry A. Foy J. W.-D. Schneider D. J. Thompson J. D. (2014). Toxicological and Pharmacokinetic Properties of QPI-1007, a Chemically Modified Synthetic siRNA Targeting Caspase 2 mRNA, Following Intravitreal Injection. Nucleic Acid Ther. 24 (4), 258–266. 10.1089/nat.2014.0489 25054518

[B118] Song J. Yi C. (2017). Chemical Modifications to RNA: A New Layer of Gene Expression Regulation. ACS Chem. Biol. 12 (2), 316–325. 10.1021/acschembio.6b00960 28051309

[B119] Song Z. Liang X. Wang Y. Han H. Yang J. Fang X. (2019). Phenylboronic Acid-Functionalized Polyamidoamine-Mediated miR-34a Delivery for the Treatment of Gastric Cancer. Biomater. Sci. 7 (4), 1632–1642. 10.1039/c8bm01385c 30720809

[B120] Sullenger B. A. Nair S. (2016). From the RNA World to the Clinic. Science 352 (6292), 1417–1420. 10.1126/science.aad8709 27313039 PMC6035743

[B121] Sullivan S. M. (1994). Development of Ribozymes for Gene Therapy. J. Invest. Dermatol. 103 (5 Suppl. l), 85s–89s. 10.1038/jid.1994.15 7963690

[B122] Sun Y. Ye X. Cai M. Liu X. Xiao J. Zhang C. (2016). Osteoblast-Targeting-Peptide Modified Nanoparticle for siRNA/microRNA Delivery. ACS Nano 10 (6), 5759–5768. 10.1021/acsnano.5b07828 27176123

[B123] Tabebordbar M. Zhu K. Cheng J. K. W. Chew W. L. Widrick J. J. Yan W. X. (2016). In Vivo Gene Editing in Dystrophic Mouse Muscle and Muscle Stem Cells. Science 351 (6271), 407–411. 10.1126/science.aad5177 26721686 PMC4924477

[B124] The Eyetech Study Group 2003). Anti-vascular Endothelial Growth Factor Therapy for Subfoveal Choroidal Neovascularization Secondary to Age-Related Macular Degeneration: Phase II Study Results*.* Ophthalmology 110 (5), 979–986. 10.1016/S0161-6420(03)00085-X 12750101

[B125] Tolcher A. W. Papadopoulos K. P. Patnaik A. Rasco D. W. Martinez D. Wood D. L. (2015). Safety and Activity of DCR-MYC, a First-In-Class Dicer-Substrate Small Interfering RNA (DsiRNA) Targeting MYC, in a Phase I Study in Patients with Advanced Solid Tumors. Jco 33 (15_Suppl. l), 11006. 10.1200/jco.2015.33.15_suppl.11006

[B126] Trepotec Z. Aneja M. K. Geiger J. Hasenpusch G. Plank C. Rudolph C. (2019). Maximizing the Translational Yield of mRNA Therapeutics by Minimizing 5'-UTRs. Tissue Eng. Part. A. 25 (1-2), 69–79. 10.1089/ten.TEA.2017.0485 29638193

[B127] Trepotec Z. Lichtenegger E. Plank C. Aneja M. K. Rudolph C. (2019). Delivery of mRNA Therapeutics for the Treatment of Hepatic Diseases. Mol. Ther. 27 (4), 794–802. 10.1016/j.ymthe.2018.12.012 30655211 PMC6453508

[B39] USFDA (2019). FDA Approves Givosiran for Acute Hepatic Porphyria. Available from: https://www.fda.gov/drugs/resources-information-approved-drugs/fdaapproves-givosiran-acute-hepatic-porphyria (Accessed March 2, 2021).

[B128] USFDA (2021a). Moderna COVID-19 Vaccine. Available from: https://www.fda.gov/emergency-preparedness-and-response/coronavirus-disease-2019-covid-19/moderna-covid-19-vaccine (Accessed March 2, 2021).

[B129] USFDA (2021b). Pfizer-BioNTech COVID-19 Vaccine. Available from: https://www.fda.gov/emergency-preparedness-and-response/coronavirus-disease-2019-covid-19/pfizer-biontech-covid-19-vaccine (Accessed March 2, 2021).

[B130] Varghese A. M. Ang C. Dimaio C. J. Javle M. M. Gutierrez M. Yarom N. (2020). A Phase II Study of siG12D-LODER in Combination with Chemotherapy in Patients with Locally Advanced Pancreatic Cancer (PROTACT). Jco 38 (15_Suppl. l), TPS4672. 10.1200/jco.2020.38.15_suppl.tps4672

[B131] Vater A. Klussmann S. (2015). Turning Mirror-Image Oligonucleotides into Drugs: The Evolution of Spiegelmer Therapeutics. Drug Discov. Today 20 (1), 147–155. 10.1016/j.drudis.2014.09.004 25236655

[B132] Vellas C. Delobel P. De Souto Barreto P. Izopet J. (2020). COVID-19, Virology and Geroscience: A Perspective. J. Nutr. Health Aging 24 (7), 685–691. 10.1007/s12603-020-1416-2 32744561 PMC7301052

[B133] Vinores S. A. (2006). Pegaptanib in the Treatment of Wet, Age-Related Macular Degeneration. Int. J. Nanomedicine 1 (3), 263–268. 17717967 PMC2426796

[B134] Wagner M. J. Mitra R. McArthur M. J. Baze W. Barnhart K. Wu S. Y. (2017). Preclinical Mammalian Safety Studies of EPHARNA (DOPC Nanoliposomal EphA2-Targeted siRNA). Mol. Cancer Ther. 16 (6), 1114–1123. 10.1158/1535-7163.mct-16-0541 28265009 PMC5457703

[B135] Wan G. Liu Y. Han C. Zhang X. Lu X. (2014). Noncoding RNAs in DNA Repair and Genome Integrity. Antioxid. Redox Signaling 20 (4), 655–677. 10.1089/ars.2013.5514 PMC3901350 23879367

[B136] Weissman D. (2015). mRNA Transcript Therapy. Expert Rev. Vaccin. 14 (2), 265–281. 10.1586/14760584.2015.973859 25359562

[B137] Weng D. E. Masci P. A. Radka S. F. Jackson T. E. Weiss P. A. Ganapathi R. (2005). A Phase I Clinical Trial of a Ribozyme-Based Angiogenesis Inhibitor Targeting Vascular Endothelial Growth Factor Receptor-1 for Patients with Refractory Solid Tumors. Mol. Cancer Ther. 4 (6), 948–955. 10.1158/1535-7163.mct-04-0210 15956252

[B138] Wojtal D. Kemaladewi D. U. Malam Z. Abdullah S. Wong T. W. Y. Hyatt E. (2016). Spell Checking Nature: Versatility of CRISPR/Cas9 for Developing Treatments for Inherited Disorders. Am. J. Hum. Genet. 98 (1), 90–101. 10.1016/j.ajhg.2015.11.012 26686765 PMC4716669

[B139] Xie F. Ye L. Chang J. C. Beyer A. I. Wang J. Muench M. O. (2014). Seamless Gene Correction of β-thalassemia Mutations in Patient-specific iPSCs Using CRISPR/Cas9 andpiggyBac. Genome Res. 24 (9), 1526–1533. 10.1101/gr.173427.114 25096406 PMC4158758

[B140] Young B. E. Kundu N. Sczepanski J. T. (2019). Mirror‐Image Oligonucleotides: History and Emerging Applications. Chem. Eur. J. 25 (34), 7981–7990. 10.1002/chem.201900149 30913332 PMC6615976

[B141] Yuen M. F. Schiefke I. Yoon J. H. Ahn S. H. Heo J. Kim J. H. (2020). RNA Interference Therapy with ARC‐520 Results in Prolonged Hepatitis B Surface Antigen Response in Patients with Chronic Hepatitis B Infection. Hepatology 72 (1), 19–31. 10.1002/hep.31008 31654573 PMC7496196

[B142] Zabludoff S. Liu Y. Liu J. Zhang J. Xia F. Quimbo A. (2017). Late Breaking Abstract - ND-L02-s0201 Treatment Leads to Efficacy in Preclinical IPF Models. Eur. Respir. J. 50 (Suppl. 61), PA881. 10.1183/1393003.congress-2017.pa881

[B143] Zelinski D. P. Zantek N. D. Stewart J. C. Irizarry A. R. Kinch M. S. (2001). EphA2 Overexpression Causes Tumorigenesis of Mammary Epithelial Cells. Cancer Res. 61 (5), 2301–2306. 11280802

